# Accuracy of laboratory tests collected at referring hospitals versus tertiary care hospitals for acute stroke patients

**DOI:** 10.1371/journal.pone.0214874

**Published:** 2019-04-10

**Authors:** Thanin Lokeskrawee, Sombat Muengtaweepongsa, Pattarapol Inbunleng, Phichayut Phinyo, Jayanton Patumanond

**Affiliations:** 1 Department of Emergency Medicine, Lampang Hospital, Muang District, Lampang, Thailand; 2 Neurology Unit, Department of Internal Medicine, Center of Excellence in Stroke, Thammasat University, Pathum Thani Thailand; 3 Department of Emergency Medicine, Lampang Hospital, Muang District, Lampang, Thailand; 4 Research Division, Maesai District Hospital, Maesai District, Chiang Rai, Thailand; 5 Center for Clinical Epidemiology and Clinical Statistics, Faculty of Medicine, Chiang Mai University, Chiang Mai, Thailand; University of Ioannina School of Medicine, GREECE

## Abstract

**Background:**

The standard treatment of acute ischemic stroke patients is thrombolytic therapy within 60 minutes of a patient’s arrival in stroke center hospitals. Based on the policy of the Lampang Referral System Committee, blood samples of suspected stroke patients need to be collected before transfer to the stroke center (Lampang Hospital). It was still questionable as to whether these blood samples are valid for clinical use and the present study aimed to confirm or deny their validity.

**Methods:**

A diagnostic study was conducted from June 2015 to May 2016. After exclusion, 340 patients were deemed eligible for analysis. Blood samples were collected just before normal saline infusion at referring hospitals and stored in blood collecting tube boxes set during transportation. At the stroke center, informed consents was requested, blood samples were re-collected to serve as a ‘gold standard’. Prothrombin time (PT), international normalized ratio (INR), activated partial thromboplastin time (aPTT), platelet count, hemoglobin (Hb), hematocrit (Hct), blood urea nitrogen (BUN), and creatinine (Cr) were compared using paired t-tests. Binary regression was used to analyze for accuracy (%) to adjust for extraneous influences and was presented by modified Bland-Altman plots.

**Results:**

The laboratory results of referring hospitals vs. the stroke center were: PT, 12.4±3.2 vs. 12.5±3.0 sec; INR: 1.0±0.3 vs. 1.0±0.3; and platelet count: 239.8±77.1 vs. 239.8±74.8 (x103/μL). The adjusted accuracy of the PT, INR, and platelet counts were 96.8%, 96.8%, and 95.3% respectively.

**Conclusion:**

Laboratory tests from referring hospital were determined to be valid. Blood samples should thus be collected at referring hospitals in order to avoid unnecessary blood collection at the stroke center.

## Introduction

In developing countries, patients with acute ischemic stroke within 3–4.5 hours of presenting at rural hospitals often need to be transferred to stroke centers at tertiary hospitals for intravenous thrombolysis (recombinant tissue plasminogen activator: rt-PA). At stroke centers, the decision for rt-PA administration requires brain imaging and laboratory tests: prothrombin time (PT), international normalized ratio (INR), and platelet counts [1]. However, laboratory tests at rural hospitals are not available 24-hours. At the Lampang Hospital (743 beds Tertiary Hospital, cover 12 rural hospitals and 2 provincial hospitals in the Northern part of Thailand), the process of obtaining blood samples in the Emergency Department may be difficult for some patients, especially the elderly who usually took 5.7±7.7 minutes [2].

Since 2010, the Lampang Referral System Committee has encouraged health care providers to collect blood samples before intravenous fluid infusion at rural hospitals, and that blood samples should be transferred with the patients to the stroke center and sent to laboratory departments as soon as the patients arrive. However, the accuracy of these laboratory tests is still debatable due to extraneous influences (preanalytical variables) such as equipment [3], phlebotomy techniques [4], needle sizes [5], tourniquet application and venous stasis [6], the order of tube filling [7], tube mixing [8], transportation [3], temperature, storage time [9], and the volume of intravenous fluid infusion [10].

Based on Clinical Laboratory Improvement Amendments (CLIA) [11–13], allowable errors vary between ±6% to ±25% depending on the types of laboratory test. The objective of this study was to assess the accuracy of laboratory tests from blood samples obtained at the referring hospitals.

## Materials and methods

### Patients and setting

A prospective diagnostic accuracy study was conducted at Lampang, in the north of Thailand during June 2015 to May 2016. Consecutive suspected acute stroke patients at the referring hospitals were given routine care and 15 ml of blood samples were collected before intravenous fluid infusion. To reduce confounding factors in blood collection, standard phlebotomy according to the Clinical and Laboratory Standards Institute (2008) [14] was followed. Blood samples were stored in blood collecting tube box sets. The time and temperature were recorded. During transportation, the temperature was recorded 15 minutes after the ambulance departed to represent the temperature inside the ambulance. At the stroke center, informed consent was requested, and blood samples were re-collected to serve as reference samples (the ‘gold standard’), accompanied by the recorded time and temperature. The blood samples from the two sites were then sent to the hematology and blood chemistry departments, where the time and temperature were recorded before the test were conducted.

The study has been approved by The Human Research Ethics Committee, Lampang Hospital and all ethics committees of the participating hospitals.

#### Blood collecting tube box sets

The blood samples were stored in the blood collecting tube box sets, which were specifically designed for this study. Within the box, spaces for tubes from referring hospitals and stroke centers were clearly labeled to avoid misplacement. The boxes were equipped with digital thermometers and digital clocks which were calibrated once a week.

### Definitions

1. Percentage error (% error): the error of the laboratory tests from referring hospitals was compared with the stroke center (the ‘gold standard’) using the equation:
  laboratory test results from the referring hospitals—the stroke center x 100 laboratory test results from the stroke center
2. Allowable error (%): the acceptable percentage errors as defined by the CLIA [11–13]:
  Prothrombin time (PT): ±15%;
  International normalized ratio (INR): ±15%;
  Activated partial thromboplastin time (aPTT): ±15%;
  Hemoglobin (Hb): ±7%;
  Hematocrit (Hct): ±6%;
  Platelet count: ±25%;
  Blood urea nitrogen (BUN): ±9%; and
  Creatinine (Cr): ±15%.
3. Crude accuracy (%): percentage of allowable error for each laboratory test before adjusting for extraneous influences.
4. Adjusted accuracy (%): percentage of allowable error for each laboratory test after adjusting for extraneous influences such as temperature, volume of intravenous fluid infusion, and storage time.
5. Storage time: the duration of time from blood collection to the time before the tests were conducted.

#### Study size estimation

Based on the pilot study, the mean INR of referring hospitals and stroke centers were 1.16±0.75 vs. 1.17± 0.75. To obtain the power of 80%, alpha error of 5%, with a two-sided test, this study required at least a total of 307 patients to validate the various laboratory tests.

### Statistical analysis

Baseline characteristics were presented by number (percentage) for categorical data and by mean±SD for numerical data.

The mean value of laboratory tests between referring hospitals and the stroke center were compared using paired t-tests, with laboratory tests having a p-value of >0.05 being interpreted as insignificant. Mean difference and percentage error were also analyzed and presented using modified Bland-Altman plots.

We calculated the crude accuracy for each laboratory test according to the CLIA standard [11–13]. The adjusted accuracy was analyzed by binary regression to adjust for the extraneous influences.

To assess whether the storage time had any effect on accuracy, we classified the storage time into three groups: under 30, 30–60, and more than 60 minutes. The difference between the three groups were tested using Wald’s tests.

## Results

**Fig 1:**
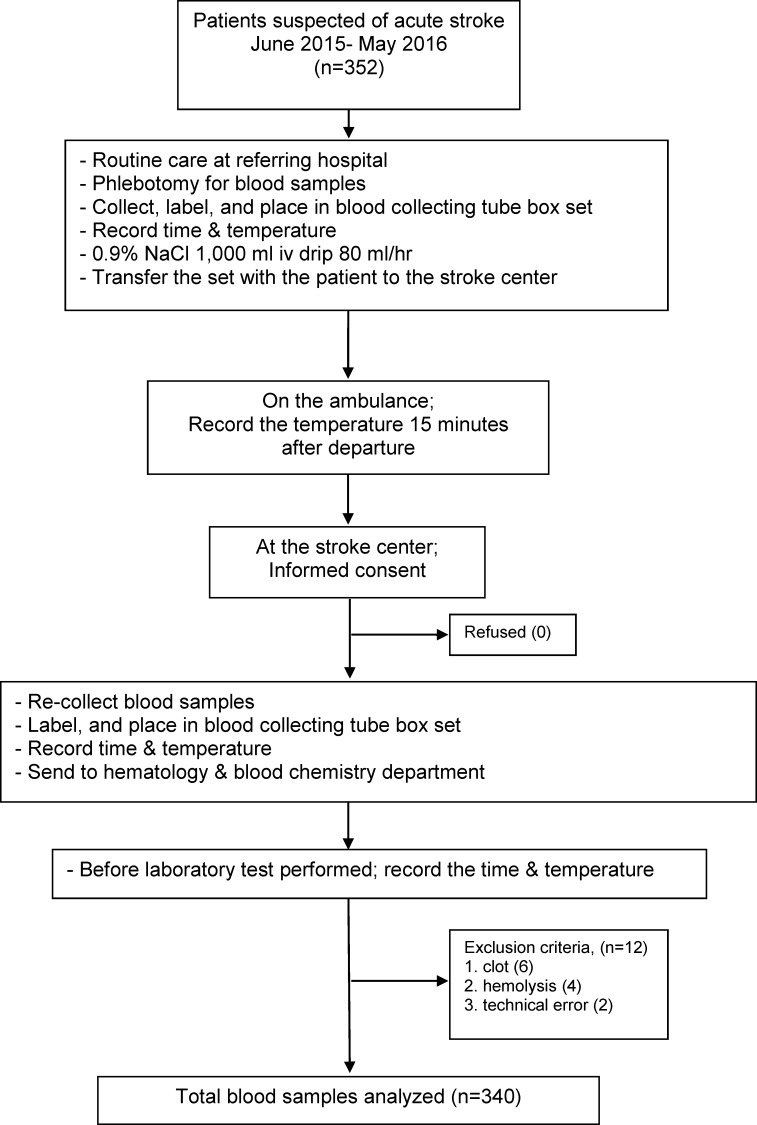
Study flow.

Between June 2015 and May 2016, there were 352 patients who met the study criteria. After exclusion (n = 12, 3.4%), 340 remaining patients were deemed to be eligible for analysis (Fig 1).

The majority of the patients were male, diagnosed with ischemic stroke. Half of the storage time was less than 30 minutes (Table 1).

**Table 1:** Baseline characteristics of patients with suspected acute stroke (n = 340).

Characteristics	n	%
Gender		
Male	188	55.3
Female	152	44.7
Age (years), mean±SD	67.4±12.7	
Final diagnosis		
Ischemic stroke	234	68.9
Hemorrhagic stroke	79	23.2
Other*	27	7.9
Storage times (minutes)		
<30	145	42.7
30–60	85	25.0
>60	110	32.3
Volume of intravenous fluid infusion (ml), mean±SD	129.0±85.9	
Temperature (degree Celsius), mean±SD		
at referring hospitals	26.9±3.6	
in ambulances	26.9±3.8	
at stroke center, emergency department	24.4±2.1	
at hematology department	24.9±2.1	
at blood chemistry department	24.7±1.3	
Storage time (minutes), mean±SD	92.1±42.6	

SD, standard deviation

*Transient ischemic attacks (TIA) and seizures

### Mean values, mean differences, and percentage errors

The mean differences of laboratory tests varied from -1.2±22.7 to 0.6±1.7 units. Most of the comparisons were significantly different (p<0.001), except for the platelet counts. (p = 0.354) (Table 2).

**Table 2:** Comparison of laboratory tests from referring hospitals vs. stroke center.

**Laboratory tests**	**Referring hospital** **(mean±SD)**	**Stroke center** **(mean±SD)**	**p-value**	**Mean of difference** **(mean±SD)**	**Error margin** **(%)**	Allowable error^*^ (%)	**Crude accuracy (%)**	**Adjusted accuracy (%)**
Prothrombin time (PT) (sec)	12.4±3.2	12.5±3.0	0.003	-0.1±0.5	-0.7±2.8	±15	96.5	96.8
International normalized ratio (INR)	1.0±0.3	1.0±0.3	0.003	-0.01±0.04	-0.7±3.0	±15	96.2	96.8
Activated partial thromboplastin time (aPTT) (sec)	25.3±3.8	24.9±3.2	<0.001	0.4±2.1	1.9±8.7	±15	88.2	89.3
Hemoglobin (Hb) (gm/dL)	12.4±2.0	12.3±2.0	<0.001	0.1±0.5	1.1±4.3	±7	86.2	89.7
Hematocrit (Hct) (vol%)	37.9±5.9	37.3±5.9	<0.001	0.6±1.7	1.7±4.5	±6	78.2	81.8
Platelet count (x10^3^/μL^3^)	240.3±76.6	240.4±74.2	0.354	-1.2±22.7	-0.4±9.1	±25	93.2	95.3
Blood urea nitrogen (BUN) (mg/dL)	19.8±18.4	19.3±18.4	<0.001	0.4±0.9	3.3±6.0	±9	81.5	81.2
Creatinine (Cr) (mg/dL)	1.4±2.0	1.3±2.0	<0.001	0.02±0.07	2.4±6.1	±15	96.8	94.6
*CLIA [10–12]; SD, standard deviation								
**Laboratory tests**	**Referring hospital** (mean±SD**)	**Stroke center** (mean±SD**)	**p-value**	**Mean of difference** (mean±SD**)	**Error margin** **(%)**	Allowable error^*^ (%)	**Crude accuracy (%)**	**Adjusted accuracy (%)**
Prothrombin time (PT) (sec)	12.4±3.2	12.5±3.0	0.003	-0.1±0.5	-0.7±2.8	±15	96.5	96.8
International normalized ratio (INR)	1.0±0.3	1.0±0.3	0.003	-0.01±0.04	-0.7±3.0	±15	96.2	96.8
Activated partial thromboplastin time (aPTT) (sec)	25.3±3.8	24.9±3.2	<0.001	0.4±2.1	1.9±8.7	±15	88.2	89.3
Hemoglobin (Hb) (gm/dL)	12.4±2.0	12.3±2.0	<0.001	0.1±0.5	1.1±4.3	±7	86.2	89.7
Hematocrit (Hct) (vol%)	37.9±5.9	37.3±5.9	<0.001	0.6±1.7	1.7±4.5	±6	78.2	81.8
Platelet count (x10^3^/μL^3^)	240.3±76.6	240.4±74.2	0.354	-1.2±22.7	-0.4±9.1	±25	93.2	95.3
Blood urea nitrogen (BUN) (mg/dL)	19.8±18.4	19.3±18.4	<0.001	0.4±0.9	3.3±6.0	±9	81.5	81.2
Creatinine (Cr) (mg/dL)	1.4±2.0	1.3±2.0	<0.001	0.02±0.07	2.4±6.1	±15	96.8	94.6

*CLIA [10–12]

**SD, standard deviation

**Fig 2:**
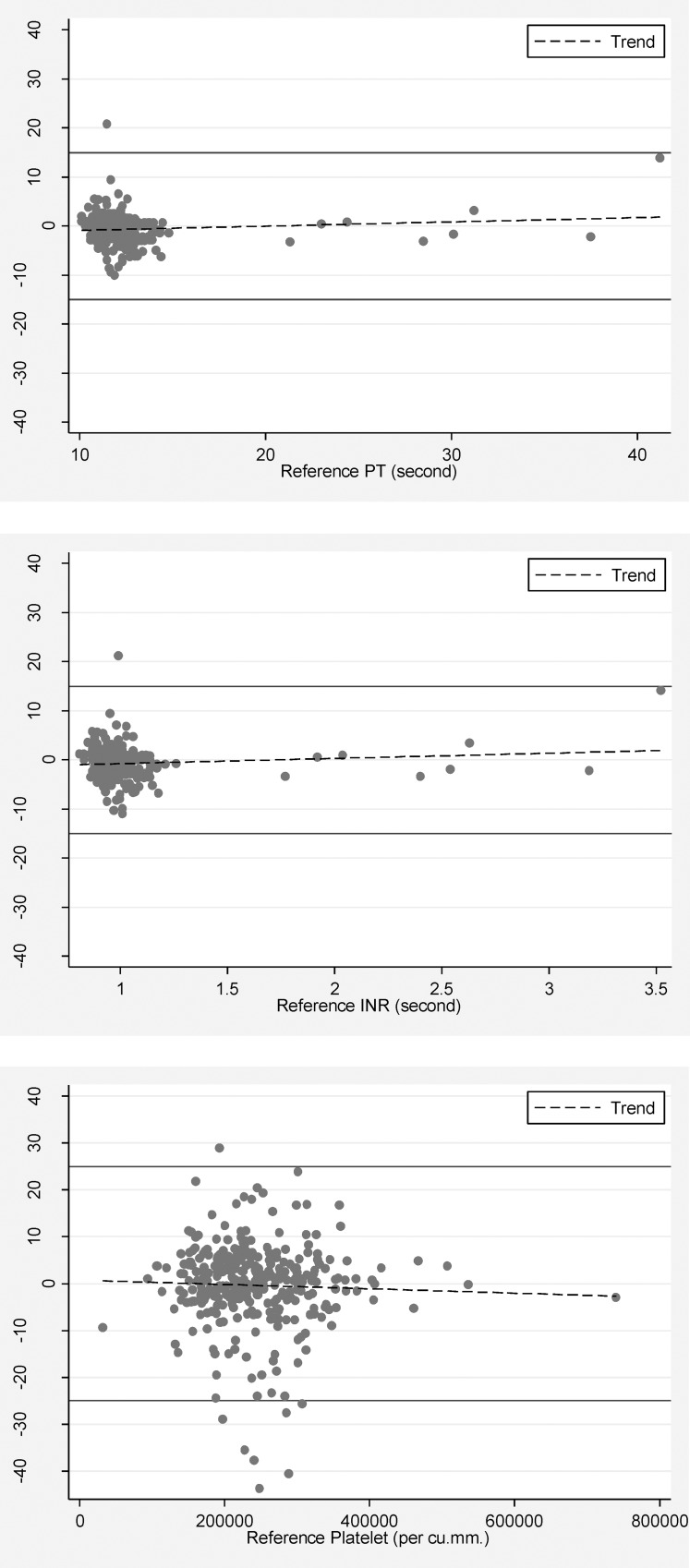
Modified Bland-Altman plots of prothrombin time (PT), international normalized ratio (INR), and platelet counts.

Percentage errors of PT, INR, and platelet counts were all within an allowable error range, according to the CLIA standard [11–13] (Fig 2).

### Accuracy

All laboratory tests from the referring hospitals had a high adjusted accuracy rate, ranging from 81.2% to 96.8%. The three highest adjusted accuracy laboratory tests were PT (96.8%), INR (96.8%), and platelet count (95.3%). The two lowest adjusted accuracy tests were BUN (81.2%), and Hct (81.8%) (Table 2). The accuracy rate was 100% in eight patients with INR > 1.7.

### The influence of storage times

The differences of adjusted accuracy classified by storage time varied from 2.2% to 7.9%, however these differences were not considered statistically significant (Table 3).

**Table 3:** Adjusted accuracy classified by storage time.

Laboratory tests	Adjusted accuracy (%) classified by storage time (min)			Range (max-min) (%)	p-value
	<30	30–60	>60		
Prothrombin time (PT)	97.7	95.3	96.4	2.4	0.561
International normalized ratio (INR)	97.7	95.3	96.4	2.4	0.561
Activated partial thromboplastin time (aPTT)	89.8	87.7	89.9	2.2	0.862
Hemoglobin (Hb)	84.7	89.6	92.6	7.9	0.264
Hematocrit (Hct)	82.6	77.7	83.7	6.0	0.805
Platelet count	95.0	93.6	96.7	3.1	0.930
Blood urea nitrogen (BUN)	84.5	78.8	79.8	5.7	0.443
Creatinine (Cr)	94.4	99.1	98.0	4.7	0.996

## Discussion

**Fig 3:**
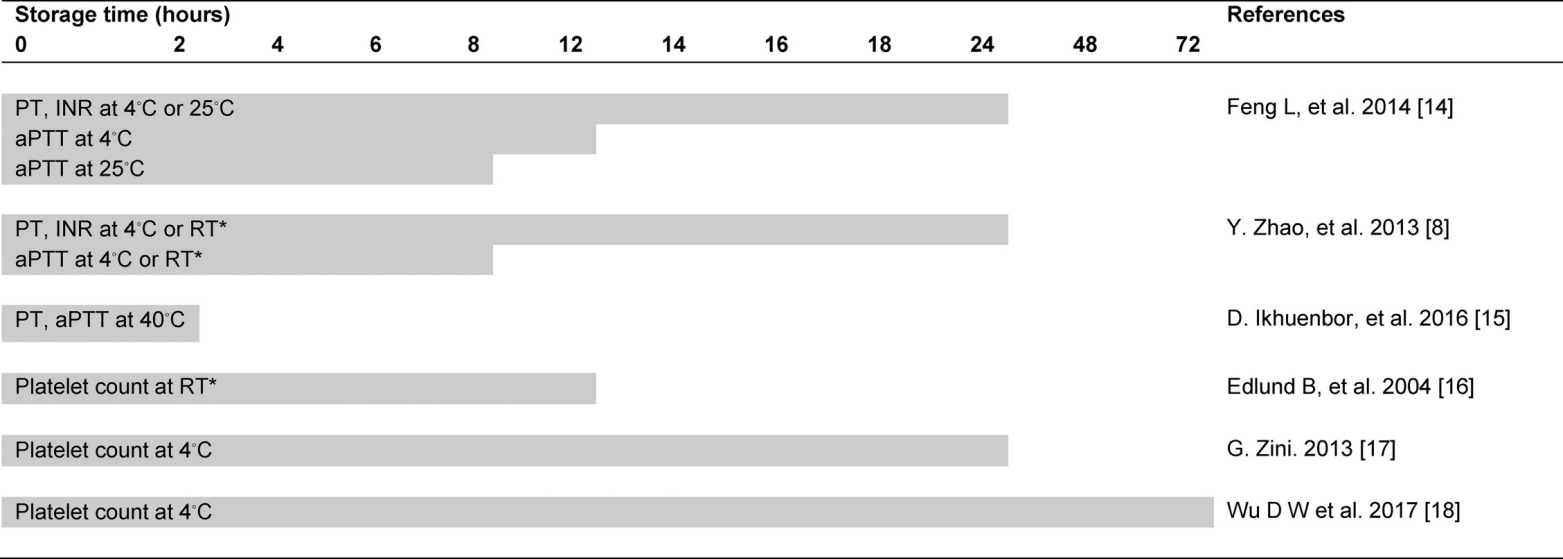
Different acceptable storage times of laboratory tests as proposed by previous researchers.

Previous studies have stated diverse acceptable storage times for blood samples, ranging from 2 to 72 hours, depending on the degree of storage temperatures. In higher storage temperature, the acceptable storage time was shorter [9, 15–19] (Fig 3). In the present study, the mean storage time was less than 2 hours (maximum 350 min) and the average storage temperatures were somewhat higher than the average room temperature (20–25 degree Celsius) which ensured the accuracy of laboratory results according to the standard.

In developing countries, until now, there have been no studies regarding the accuracy of using blood samples collected from the referring hospitals as a reference for treatment at the stroke center. In the absence of any evidence, blood recollection at the stroke center is still common. Integrating the art of clinical science and clinical practice, this study may be the first to address this laboratory dilemma. The strength of this study is that multiple factors were taken into consideration during data analysis to ensure the validity of the laboratory results.

Apart from PT, INR, and platelet counts, this study also suggests the accuracy of other laboratory tests, such as Cr. With a high level of adjusted accuracy (94.6%), this test value could be applied to other referral settings, for example, for emergency patients requiring computed tomography with radiocontrast agents in which the renal function need to be first evaluated.

Other laboratory values had lower accuracy rates, notably Hb and Hct levels, which could be affected by various external influences, such as undetected red cell hemolysis, unstable blood storage, and other unrecognized factors.

Referring patients to stroke centers from various district hospitals takes different duration of time depending on distance, traffic, and road conditions. By stratifying storage time into three groups (<30, 30–60, and >60 minutes), this study showed that the adjusted accuracy of the laboratory results did not vary, which ensures applicability in clinical practice.

A 2018 guidelines for the early management of patients with acute ischemic stroke by American Heart Association / American Stroke Association stated that in patients with clearly no current use of warfarin, non-vitamin K antagonist (NOAC) or heparin, treatment with intravenous thrombolysis should be able to start prior to present of PT/INR or aPTT results but needs be stopped when INR is over 1.7

[20]. Nevertheless, up to the present, Thai Stroke Society still requires hematologic exams (PT, INR, and platelet count) before thrombolytic agent administration.

There were some other laboratory tests which were not included in this study, such as blood electrolytes, which fluctuate according to time, intravenous fluid infusion, and disease progression. However, in acute stroke patient care, these laboratory tests are outside our area of concern.

## Conclusions

Blood samples collected from referring hospitals are valid and should be used instead of requiring unnecessary blood collection at the stroke center. The accuracy rate remained valid even when the storage time was delayed up to 350 minutes.
